# Effects of inflammatory cytokine IL-27 on the activation of fibroblast-like synoviocytes in rheumatoid arthritis

**DOI:** 10.1186/ar3067

**Published:** 2010-07-06

**Authors:** Chun K Wong, Da P Chen, Lai S Tam, Edmund K Li, Yi B Yin, Christopher WK Lam

**Affiliations:** 1 Department of Chemical Pathology, The Chinese University of Hong Kong, Prince of Wales Hospital, Ngan Shing Street, Shatin, NT, Hong Kong; 2 Department of Medicine and Therapeutics, The Chinese University of Hong Kong, Prince of Wales Hospital, Ngan Shing Street, Shatin, NT, Hong Kong; 3 Key Laboratory of Diagnostic Medicine designated by the Ministry of Education, Chongqing Medical University, Yu Zhong Qu, Yi Xue Yuan Lu, Chongqing, 400016, PR China; 4 Macau Institute for Applied Research in Medicine and Health, Macau University of Science and Technology, Avenida Wai Long, Taipa, Macau

## Abstract

**Introduction:**

Interleukin (IL)-27 is a novel member of the IL-6/IL-12 family cytokines that are produced early by antigen-presenting cells in T helper (Th)1-mediated inflammation. Elevated expression of IL-27 has been detected in the synovial membranes and fluid of rheumatoid arthritis (RA).

**Methods:**

We investigated the *in vitro *effects of IL-27, alone or in combination with inflammatory cytokine tumor necrosis factor (TNF)-α or IL-1 β on the pro-inflammatory activation of human primary fibroblast-like synoviocytes (FLS) from RA patients and normal control subjects, and the underlying intracellular signaling molecules were determined by intracellular staining using flow cytometry.

**Results:**

Significantly higher plasma concentration of IL-27 was found in RA patients (*n *= 112) than control subjects (*n *= 46). Both control and RA-FLS constitutively express functional IL-27 receptor heterodimer, gp130 and WSX-1, with more potent IL-27-mediated activation of signal transducers and activators of transcription (STAT)1 in RA-FLS. IL-27 was found to induce significantly higher cell surface expression of intercellular adhesion molecule (ICAM)-1 and vascular cell adhesion molecule (VCAM)-1 and release of inflammatory chemokine IL-6, CCL2, CXCL9, CXCL10 and matrix metalloproteinase-1 of RA-FLS than that of control FLS (all *P *< 0.05). Moreover, an additive or synergistic effect was observed in the combined treatment of IL-27 and TNF-α or IL-1 β on the surface expression of ICAM-1 and VCAM-1 and the release of CXCL9 and CXCL10 of RA-FLS. Further investigations showed that the expression of ICAM-1, VCAM-1 and chemokines stimulated by IL-27 was differentially regulated by intracellular activation of phosphatidylinositol 3-OH kinase-AKT, c-Jun amino-terminal kinase and Janus kinase pathways.

**Conclusions:**

Our results therefore provide a new insight into the IL-27-activated immunopathological mechanisms mediated by distinct intracellular signal transductions in joint inflammation of RA.

## Introduction

Rheumatoid arthritis (RA) is a chronic autoimmune disease with 1% prevalence in the industrialized countries, characterized by cytokine-mediated inflammation of the synovial lining of the diarthrodial joints and the destruction of cartilage and bone [1]. Together with T and B lymphocytes and macrophages, fibroblast-like synoviocytes (FLS) play crucial roles in both joint damage and the propagation of inflammation in RA [2]. Normal synovial tissue consists of two anatomically distinct layers: a surface layer (intima or synovial lining) and an underlying layer (subintima). The predominant cell types in the normal intima and subintima are macrophage-like (type A) synoviocytes and FLS (type B synoviocytes). FLS are bipolar, spindle-shaped cells with prominent secretory machinery, including extensive endoplasmic reticulum, regular ribosomal arrays and well-developed Golgi apparatus [2]. FLS can mediate cartilage and bone destruction in RA mainly via the elucidation of matrix metalloproteinases (MMPs) such as MMP-1 [3]. Additionally, FLS can secrete receptor activators of nuclear factor-κB ligand (RANKL) that attract macrophages from the vasculature; stimulate the differentiation of vascular- and tissue-derived macrophages into osteoclasts; and activate osteoclasts at the bone surface, leading to bone erosion [4].

FLS mediates inflammation and autoimmunity through a wide range and complex mechanisms during the development of RA [2]. In RA, FLS respond to inflammatory cytokines including interleukin (IL)-1β, 6, 8, 12, 17, 18, 21, tumor necrosis factor (TNF)-α and interferon (IFN)-γ through the activation of multiple intracellular signaling pathways including extracellular signal-regulated protein kinase (ERK), c-Jun amino-terminal kinase (JNK) and p38 mitogen activated protein kinase (MAPK), leading to the expression of multiple cytokines such as IL-1β, IL-6, and TNF-α, as well as the secretion of MMPs that contribute to tissue destruction [3,5-8]. Adhesion molecules present on the FLS surface, including CD44, vascular cell adhesion molecule (VCAM), and intercellular adhesion molecule (ICAM), regulate the trafficking of leukocytes into and/or through the synovial tissue [9]. Direct contact between FLS and T cells can induce T cell activation [10]. Secretion of IL-15 from FLS, along with the other cytokines, activates T cells, neutrophils and macrophages [10].

IL-27 is a heterodimeric cytokine composed of EBV-induced protein 3 (EBI3) and p28 subunit that signals through a receptor complex composed of the unique IL-27Rα (IL-27R) (WSX-1/T cell cytokine receptor (TCCR)) and gp130 signaling subunit. IL-27 belongs to IL-6/IL-12 family of structurally related ligands that include IL-12, IL-23 and IL-6 [11,12]. IL-27 receptor has been found to be expressed on naïve T cells, NK cells, monocytes, mast cells, activated B cells, Langerhan's cells and activated dendritic cells, whereas IL-27 is produced mainly by activated macrophages and dendritic cells [11,12]. IL-27 can induce a T helper (Th) type 1 response and the expression of T-bet and interferon (IFN)-γ by naïve T cells [11,13]. In innate immunity, IL-27 can provoke the production of IL-1β, TNF-α, IL-18 and IL-12 in monocytes [14]. However, more recent studies revealed that IL-27 can play a regulatory role by suppressing the acquired immunity, inducing the development of Th cells, and expansion of inducible regulatory T cells to produce IL-10 [11,15-17]. However, the molecular basis for pleiotropic actions of IL-27 in various immune responses has not been well elucidated yet. In animal studies of RA, administration of IL-27 can attenuate collagen-induced arthritis at the disease onset stage in mice [17]. However, in clinical studies, elevated protein and gene expression IL-27 has been detected in the established RA synovial membranes and fluid [17]. Moreover, IL-27 can induce a Th1 immune response and susceptibility to proteoglycan-induced arthritis in murine model [18]. Consistent with these findings, adjuvant-induced arthritis in rats and experimental autoimmune encephalomyelitis in mice can be abolished by anti-IL-27 antibodies [19]. Therefore, the immunopathological roles of IL-27 on the activation of FLS in RA remained unsettled, particular at the local inflammatory joints. In an attempt to further elucidate the immunopathological roles of IL-27 in the joint inflammation of RA, the *in vitro *activating effect of IL-27 in combination with TNF-α or IL-1β on FLS from RA and control subjects and its underlying intracellular signal mechanism was investigated in this study.

## Materials and methods

### RA patients, control subjects and blood samples

Patients with active RA (*n *= 112), who fulfilled the American College of Rheumatology 1987 criteria for RA [20], were enrolled in this study. Patients eligible for the study included those with active RA despite treatment with at least 12.5 mg of methotrexate per week. Active RA was defined by the presence of at least four swollen and tender joints and in addition at least two of the following: morning stiffness lasting at least 45 minutes, erythrocyte sedimentation rate (ESR) Westegren of at least 28 mm/h, and a serum C-reactive protein concentration of at least 20 mg/L [21]. Forty-six sex- and age-matched healthy Chinese volunteers were recruited as control subjects. Six mL of EDTA venous peripheral blood were collected from each patient and control subject. The above protocol was approved by the Clinical Research Ethics Committee of The Chinese University of Hong Kong-New Territories East Cluster Hospitals, and informed consent was obtained from all participants according to the Declaration of Helsinki.

### Reagents

Recombinant human IL-27 and TNF-α were purchased from R & D Systems, Minneapolis, MN, USA. ERK inhibitor PD98059, JNK inhibitor SP600125, p38 MAPK inhibitor SB203580, phosphatidylinositol 3-OH kinase (PI3K) inhibitor LY294002 and Janus kinase (JAK) inhibitor AG490 were purchased from Calbiochem Corp, San Diego, CA, USA. SB203580 was dissolved in water while PD98059, LY294002, SP600125 and AG490 were dissolved in dimethyl sulfoxide (DMSO). In all studies, the concentration of DMSO was 0.1% (vol/vol).

### Endotoxin-free solutions

Cell culture medium was purchased from Cell Applications Inc., San Diego, CA, USA, free of detectable lipopolysaccharide (< 0.1 EU/ml). All other solutions were prepared using pyrogen-free water and sterile polypropylene plastic ware. No solution contained detectable LPS, as determined by the Limulus amoebocyte lyase assay (sensitivity limit 12 pg/ml; Biowhittaker Inc, Walkersville, MD, USA).

### Cell culture of FLS

Human FLS isolated from synovial tissues obtained from normal healthy subjects and patients with RA were purchased from Cell Applications. FLS was cultured in synoviocyte growth medium including 10% synoviocyte growth supplement (Cell Applications) in 5% CO_2 _- 95% humidified air at 37 C [22].

### Quantitative PCR of GP-130 and WSX-1 gene expression

Total RNA of FLS was extracted using RNeasy Mini Kit (Qiagen Inc., Valencia, CA, USA). All RNA samples were pre-treated with deoxyribonuclease I (Invitrogen Corp., Grand Island, CA, USA) and then stored at -70°C. For each treatment, approximately 1 μ g of total RNA was reversely transcribed to complementary DNA (cDNA) with TaqMan Reverse Transcription Reagents (Applied Biosystems Inc., Foster City, CA, USA). The mRNA expression of GP-130, WSX-1 and GAPDH (endogenous control) was quantified by real-time PCR using SYBR Green probe (Roche Diagnostics Corp., Indianapolis, IN, USA) with the use of Applied Biosystems 48-well StepOne™ Real Time PCR System. The primers human GP-130, WSX-1 and GAPDH were as follows, human hgp130 forward: 5'-TCTGGGAGTGCTGTTCTGCTT-3', human gp130 reverse: 5'-TGTGCCTTGGAGGAGTGTGA-3'; human wsx-1 forward: 5'-GCTCCTGCCTCTATGTTGGC-3', human wsx-1 reverse: 5'-CCTTCATGTTCTTGGACCAGC-3'; GAPDH forward: 5'-ATGGGGAAGGTGAAGGTCG-3', GAPDH reverse: 5'-GGGGTCATTGATGGCAACAATA-3'. Real-time PCR was performed in a 25 L reaction mixture containing primers, FastStart Universal SYBR Green master (ROX) reagent (Roche) and cDNA sample in duplicate. A negative PCR control without template and a positive PCR control (GAPDH) with a template of known amplification cycle were included. The real-time PCR reaction was performed with 95°C for 15 sec to denature cDNA, 60°C for 60 sec to allow the SYBR green probe and primers to anneal to the denatured cDNA. The cycles were repeated 40 times after an initial 10-minute denaturation at 95°C. The threshold cycle (Ct) is the PCR cycle at which an increase in reporter signal above the baseline signal can first be detected. The relative GP-130 and WSX-1 mRNA expression (*n *= 3) was obtained by comparing with the relative expression of GAPDH using 2^-ΔCt (Ct,gp130/wsx-1 - Ct,GAPDH)^.

### Western blot analysis

Cells were washed, lysed, and an equal amount of proteins to ensure equal protein loading was subjected to SDS-PAGE and then blotted onto PVDF membrane (GE Healthcare Corp, Piscataway, NJ, USA). The membrane was blocked with 5% skimmed milk and probed with primary antibody against WSX-1 at 4°C overnight. After washing, the membrane was incubated with secondary antibody coupled to horseradish peroxidase (GE Healthcare) for one hour at room temperature. Antibody-antigen complexes were then detected using an ECL chemiluminescent detection system (GE Healthcare).

### Assay of human cytokines, chemokines and MMP-1

Plasma concentration of IL-27 was quantitated by ELISA reagent from R & D Systems. The concentration of chemokine CXCL9, CXCL10, CCL2 and CCL5 in culture supernatant with equal cell number loading was measured simultaneously by bead-based multiplex cytokine assay with BD cytometric bead array (CBA) (BD Biosciences Pharmingen, San Diego, CA, USA) using a four-color FACSCalibur flow cytometer (BD Biosciences Corp, San Jose, CA) [23]. Human MMP-1 in culture supernatant was assayed by ELISA reagent (RayBiotech Inc. Norcross, GA, USA).

### Flow cytometry of adhesion molecules and gp130 on cell surface

Cells were resuspended in cold PBS and blocked with 2% human pooled serum at room temperature for 15 minutes, followed by washing with PBS, cells were incubated with the FITC-conjugated mouse anti-human ICAM-1 or VCAM-1 monoclonal antibodies, or FITC-conjugated mouse IgG1 isotypic control (BD Pharmingen) at 4°C in dark for 45 minutes. For the detection of surface expression of gp130, unconjugated mouse-anti-human gp130 (R & D Systems) followed by secondary FITC-conjugated anti-mouse IgG antibodies (BD Pharmingen) were used for the staining. After washing, cells were fixed with 1% paraformaldehyde in PBS. Expression of cell surface adhesion molecules and gp130 on 5,000 viable cells was then quantitatively analyzed by flow cytometry (FACSCalibur flow cytometer, BD Biosciences) in terms of mean fluorescence intensity (MFI).

### Intracellular staining of activated (phosphorylated) signaling molecules

The intracellular expression of phosphorylated signaling molecules was determined using previously established intracellular staining assay [23]. Briefly, cells were fixed with pre-warmed BD Cytofix Buffer (4% paraformaldehyde) for 10 minutes at 37°C after stimulation by IL-27. After centrifugation, cells were permeabilized in ice-cold methanol for 30 minutes and then stained with mouse anti-human phosphorylated AKT, phosphorylated JAK2, phosphorylated JNK, phosphorylated signal transducers and activators of transcription (STAT)1(pY701) (BD Pharmingen) and phosphorylated PI3K (Cell Signaling Technology, Beverly, MA, USA) monoclonal antibodies or mouse IgG1 isotypic control (BD Pharmingen) for 60 minutes followed by FITC conjugated goat anti-mouse secondary antibody (BD Pharmingen) for another 45 minutes at 4°C in the dark. Cells were then washed, resuspended and subjected to analysis. Expression of intracellular phosphorylated signaling molecules of 5,000 viable cells was analyzed by flow cytometry (FACSCalibur, BD Biosciences) as MFI.

### Statistical analysis

Plasma concentrations of IL-27 were expressed as median (interquartile range, IQR) as they were not in Gaussian distribution and its difference between RA and control groups was assessed by Mann-Whitney rank-sum test. The statistical significance of differences of other parameters was determined by one-way ANOVA. The values were expressed as mean ± SD from three independent experiments. Any difference with a *P-*value less than 0.05 was considered significant. When ANOVA indicated a significant difference, the Bonferroni post hoc test was then used to assess the difference between groups. All analyses were performed using the Statistical Package for the Social Sciences (SPSS) statistical software for Windows, version 16.0 (SPSS Inc, Chicago, IL, USA).

## Results

### Plasma concentration of IL-27 in RA

The recruited RA patients (female:male = 97:15) cohort was found to have mean age of 53 ± 9 (range 31 to 83 years) while control subjects (female:male = 40:6) have a mean age of 47 ± 7 (range 25 to 62 years). Plasma concentration of IL-27 was found to be significant higher in RA patients than that in control subjects (11.7 (7.2 to 19.2) vs 7.1 (4.9 to 10.0) ng/ml, *P *< 0.001).

### FLS express functional IL-27 receptor

**Figure 1:**
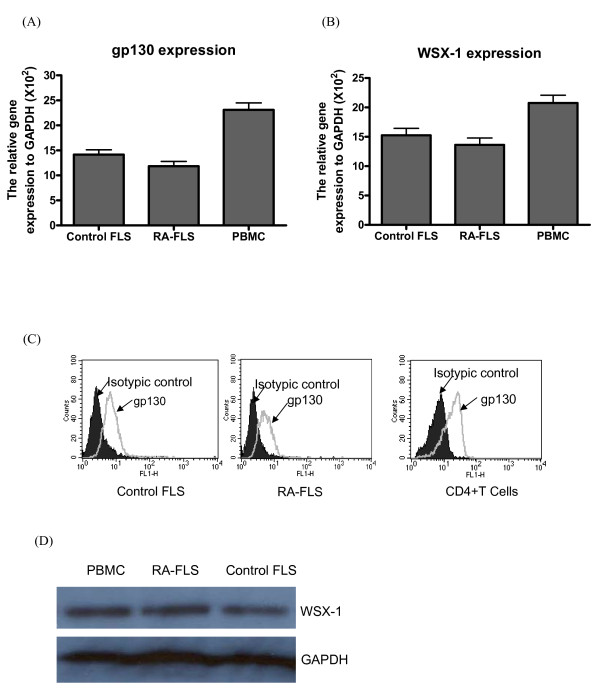
**Expression of functional IL-27 heterodimeric receptor on FLS. (A, B)**. Total RNA was extracted from control FLS, RA-FLS and PBMC (positive control), followed by quantitiatve real time RT-PCR analysis for gp130, WSX-1 and GAPDH (house keeping gene) expression. **(C) **Representative histograms of cell surface expression of gp130 on control FLS, RA-FLS and human CD4+T cells (positive control) determined by flow cytometry. **(D) **Representative Western blot analysis of WSX-1 protein expression of control FLS, RA-FLS and PBMC (positive control). GAPDH was used as protein control to ensure an equal amount of loaded protein. All the experiments were performed in three independent replicates. M, 100 base-pair molecular size marker; PBMC, peripheral blood mononuclear cells.

In view of the elevated plasma concentration of IL-27 in RA patients, we then assessed the expression of IL-27 receptors and the effect of IL-27 on FLS from RA and control subjects. We first examined the gene expression of IL-27 receptor complex, gp130 and WSX-1, of FLS while PBMC were used as positive cell control. Quantitative real time PCR analysis showed that gp130 and WSX-1 mRNA was highly expressed in control and RA-FLS, and PBMC (Figure 1A, B). Consistent with mRNA level, flow cytometric analysis showed that gp130 constitutively expressed on control and RA-FLS, and human CD4 + T cells (positive control) (Figure 1C). Because of the lack of commercial available anti-human WSX-1 antibody for flow cytometry, we confirmed the protein expression of WSX-1 of RA and control FLS using Western blot with PBMC as positive cell control (Figure 1D).

### IL-27 up-regulated ICAM-1 and VCAM-1 expression on the cell surface of FLS

**Figure 2:**
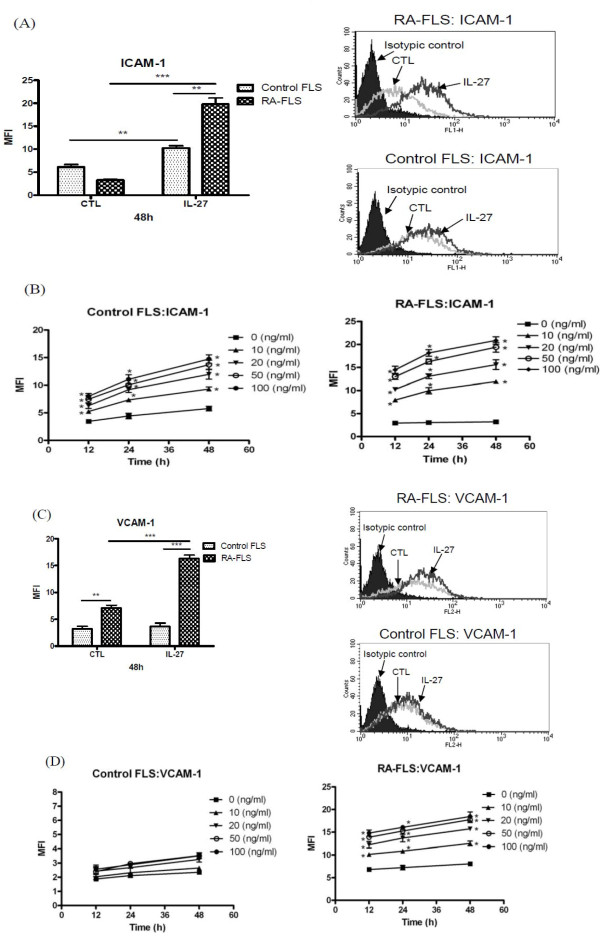
**Effect of IL-27 on the cell surface expression of adhesion molecules on control and RA-FLS. (A, C)**. Control and RA-FLS were cultured with IL-27 (50 ng/ml) for 48 h and then the surface expression of (A) ICAM-1 and (C) VCAM-1 was analysed by flow cytometry. The modulation of surface expression of adhesion molecules are shown in bar chart as MFI. Results have been normalized by subtracting appropriate isotypic control and are expressed as the arithmetic mean plus SD of three independent experiments. Representative histograms of cell surface expression of (A) ICAM-1 and (C) VCAM-1 on control FLS and RA-FLS were determined by flow cytometry. **(B, D) **Control and RA-FLS were stimulated with IL-27 (0 to 100 ng/ml) for 12 to 48 h and then the surface expression of (B) ICAM-1 and (D) VCAM-1 was analysed by flow cytometry. Results have been normalized by subtracting appropriate isotypic control and are expressed as the arithmetic mean SD of MFI from triplicate experiments. CTL: control, **P < 0.01, ***P < 0.001.

Figure 2A, C show that IL-27 (50 ng/ml) could induce significantly higher cell surface expression of ICAM-1 and VCAM-1 on RA-FLS than control FLS at 48 h (all *P *< 0.01). In the kinetics and dose response of IL-27-inducing effects on the surface expression of ICAM-1 and VCAM-1, we found that IL-27 (10 to 100 ng/ml) could significantly up-regulate the surface expression of ICAM-1 at all the incubation times (12 h to 48 h) in a dose and time dependent manner (Figure 2B, D). Furthermore, IL-27 induced significantly higher expressions of ICAM-1 and VCAM-1 on RA-FLS than that of control FLS (Figure 2B, D). In view of these results, we mainly used the optimal incubation time (48 h) and dose (50 ng/ml) of IL-27 in the following studies.

### IL-27 could enhance CCL2, CXCL9, CXCL10 and MMP-1 production from FLS

**Figure 3:**
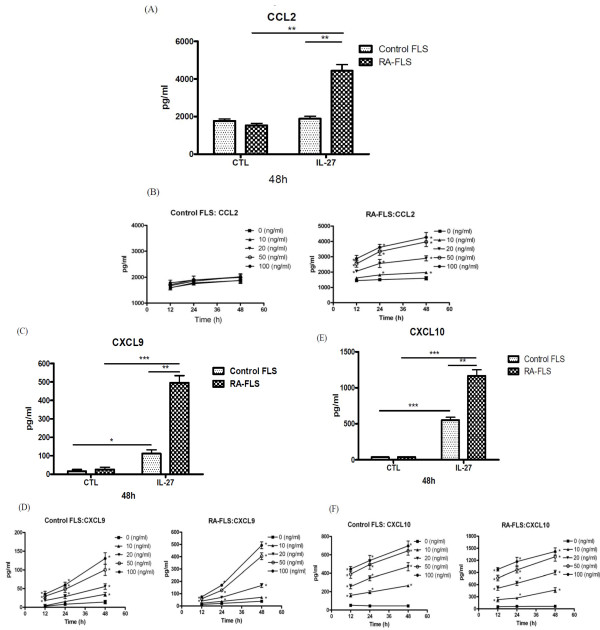
**Dose- and time-dependent effect of IL-27 on the induction of chemokine release from control and RA-FLS**. Control and RA-FLS were cultured with IL-27 (50 ng/ml) for 48 h and induction of **(A) **CCL2, **(C) **CXCL9 and **(E) **CXCL10 was analysed by CBA using flow cytometry. **(B, D, F) **Control and RA-FLS were stimulated with IL-27 (0 to 100 ng/ml) for 12 to 48 h and inductions of (B) CCL2, (D) CXCL9 and (F) CXCL10 were analysed by CBA using flow cytometry. *P < 0.05, **P < 0.01, ***P < 0.001.

As shown in Figure 3, IL-27 could induce significantly higher release of inflammatory chemokine CCL2, CXCL9 and CXCL10 from RA-FLS than that of control FLS (all *P *< 0.05). In the kinetics and dose effect of IL-27-inducing chemokine release, we found that IL-27 (10 to 100 ng/ml) could significantly induce the release of chemokines at all the incubation times (12 to 48 h) in a dose and time dependent manner. IL-27 a induced significantly higher amount of chemokines from RA-FLS than that of control FLS (Figure 3).

Furthermore, IL-27 (50 ng/ml) could induce MMP-1 (13.8 ng/ml) release comparing with medium control (9.4 ng/ml) from RA-FLS but IL-27 (10 to 100 ng/ml) cannot induce any MMP-1 release from control FLS.

### Enhanced up-regulation of adhesion molecules and the release of chemokines upon combined treatment of IL-27 and TNF-α or IL-1β

**Figure 4:**
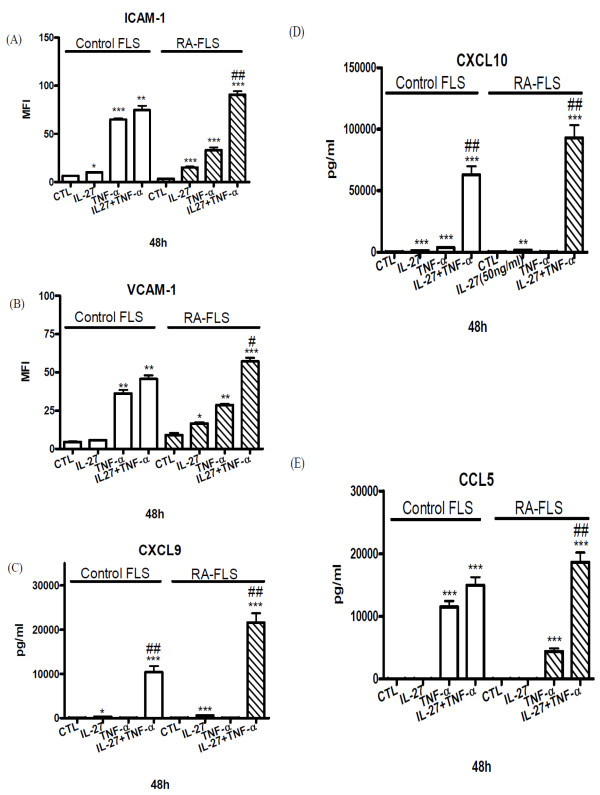
**The combined effects of IL-27 and TNF-α on the induction of adhesion molecules and chemokines. (A, B)**. Control and RA-FLS were cultured with or without IL-27 (50 ng/ml), and TNF-α (10 ng/ml) alone or in combination for 48 h. Cell surface expression of (A) ICAM-1 and (B) VCAM-1 was determined by flow cytometry. **(C, D, E) **Control and RA-FLS were cultured with or without IL-27 (50 ng/ml), and TNF-α (10 ng/ml) alone or in combination for 48 h. Release of (C) CXCL9, (D) CXCL10 and (E) CCL5 was determined by CBA using flow cytometry. Results are expressed as the arithmetic mean plus SD of three independent experiments. **P < 0.01, ***P < 0.001 when compared between treatment group and medium control. #*P *< 0.05, ##*P *< 0.01 when compared between combined treatment group and single treatment group.

**Figure 5:**
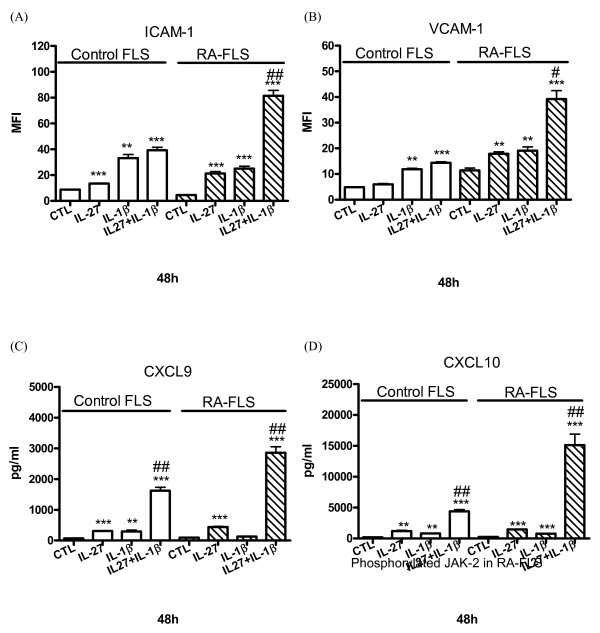
**The combined effects of IL-27 and IL-1β on the induction of adhesion molecules and chemokines. (A, B)**. Control and RA-FLS were cultured with or without IL-27 (50 ng/ml), and IL-1 (20 ng/ml) alone or in combination for 48 h. Cell surface expression of (A) ICAM-1 and (B) VCAM-1 was determined by flow cytometry. **(C, D) **Control and RA-FLS were cultured with or without IL-27 (50 ng/ml), and IL-1 (20 ng/ml) alone or in combination for 48 h. Release of (C) CXCL9 and (D) CXCL10 was determined by CBA using flow cytometry. Results are expressed as the arithmetic mean plus SD of three independent experiments. **P < 0.01, ***P < 0.001 when compared between treatment group and medium control. #*P *< 0.05, ##*P *< 0.01 when compared between combined treatment group and single treatment group.

As pro-inflammatory cytokine TNF-α and IL-1β play crucial inflammatory roles in the joints of patients [24], we further investigate the combined effect of IL-27, TNF-α and IL-1β on the activation of FLS. Figures 4 and 5 show that the combined treatment of IL-27 and TNF-α or IL-1β resulted in additive or synergistic up-regulation of ICAM-1 and VCAM-1 expression and the release of CXCL9 and CXCL10 of RA-FLS (all *P *< 0.01). Moreover, combined treatment of IL-27 and TNF-α could also exhibit synergistic induction of CCL5 from RA-FLS, and CXCL9 and CXCL10 from control FLS (both *P *< 0.05).

### Effects of IL-27 on the activation of STAT1, JAK-2, AKT, PI3K and JNK signaling pathways in FLS

**Figure 6:**
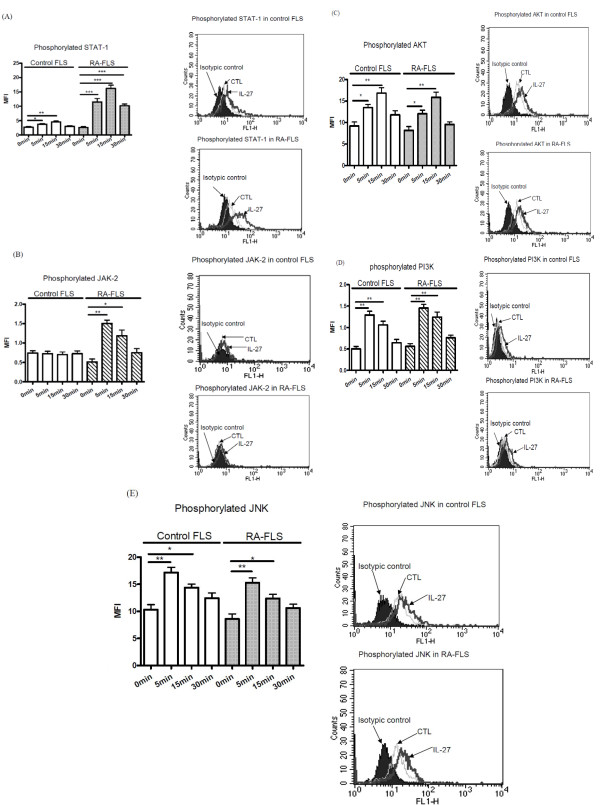
**Effects of IL-27 on intracellular STAT-1, JAK-2, AKT, JNK and PI-3K activities in FLS**. Control or RA-FLS were incubated with IL-27 (50 ng/ml) from 0 to 30 minutes. The amounts of intracellular phosphorylated signaling molecules in 5,000 permeabilized cells were measured by flow cytometry. Results of **(A) **phosphorylated STAT-1, **(B) **phosphorylated JAK-2, **(C) **phosphorylated AKT, **(D) **phosporylated PI3K and **(E) **phosphorylated JNK are shown in MFI subtracting corresponding isotypic control and are expressed as the arithmetic mean plus SD of three independent experiments. Representative histograms illustrate the intracellular expression of (A) phosphorylated STAT-1, (B) phosphorylated JAK-2, (C) phoshorylated AKT, (D) phosphorylated PI3K and (E) phosphorylated JNK in control or RA-FLS. The isotypic control represents the cell populations stained with anti-mouse IgG1 isotype control. *P < 0.05, **P < 0.01 and ***P < 0.001 when compared between groups denoted by horizontal lines.

Using intracellular fluorescence staining by flow cytometry, we measured the MFI of phosphorylated STAT1, JAK-2, AKT, PI3K, and JNK in permeabilized FLS upon IL-27 stimulation. IL-27 could induce significant phosphorylation of STAT1, AKT, PI3K and JNK in both RA and control FLS (Figure 6A, C, D, E), and JAK-2 in RA-FLS (Figure 6B), all within five minutes. IL-27 could induce significantly higher phosphorylation of STAT1 in RA-FLS than that of control FLS (Figure 6A).

### Effects of signaling molecule inhibitors on the expression of adhesion molecules and release of chemokines from FLS activated by IL-27

**Figure 7:**
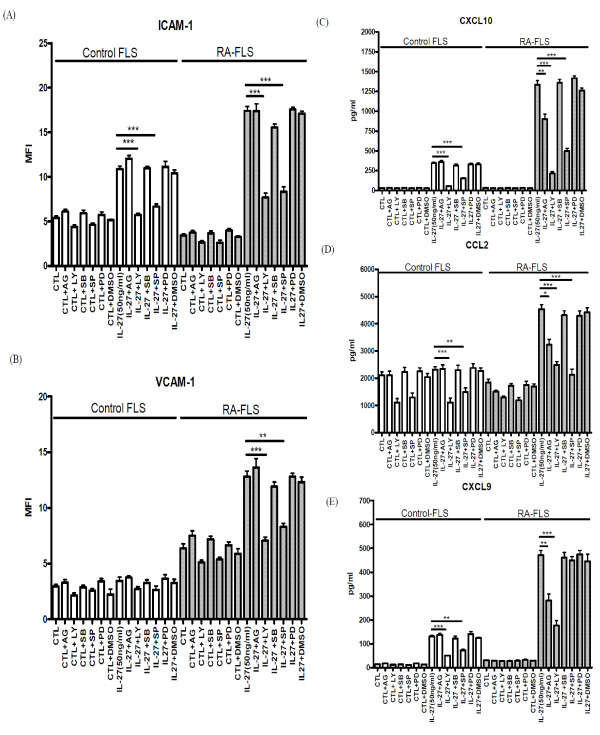
**Effects of signaling molecule inhibitors on IL-27-induced expression of adhesion molecule and chemokines of control and RA-FLS**. Control and RA-FLS were pretreated with AG490 (AG, 10 μM), LY294002 (LY, 10 μM), SB203580 (SB, 20 μM), SP600125 (SP, 10 μM) or PD98059 (PD, 10 μM) for one hour followed by incubation with or without IL-27 (50 ng/mL) in the presence of inhibitors for further 48 h. Surface expression of **(A) **ICAM-1 and **(B) **VCAM-1 of 5,000 cells was assessed by flow cytometry as MFI. Release of **(C) **CXCL10, **(D) **CCL2 and **(E) **CXCL9 was determined by CBA using flow cytometry. Results are expressed as the arithmetic mean plus SD from three independent experiments. DMSO (0.1%) was used as the vehicle control. **P < 0.01, ***P < 0.001 when compared between groups denoted by horizontal lines.

The cytotoxicities of different signaling molecule inhibitors on FLS were first determined by MTT assay. We used the optimal concentrations of AG490 (10 μM), LY294002 (10 μM), PD98059 (10 μM), SB203580 (20 μM), and SP600125 (10 μM) with significant inhibitory effects without any cell toxicity. As shown in Figure 7, PI3K inhibitor LY294002 and JNK inhibitor SP600125 could significantly suppress IL-27-induced ICAM-1 expression on the cell surface of control FLS, and IL-27-induced ICAM-1 and VCAM-1 on RA-FLS (*P *< 0.05). Regarding chemokine release, PI3K inhibitor LY294002 and JNK inhibitor SP600125 could suppress IL-27 induced CCL2, CXCL9 and CXCL10 from control FLS, JAK inhibitor AG490, PI3K inhibitor LY294002 and JNK inhibitor SP600125 could suppress IL-27 induced CCL2 and CXCL10 release from RA-FLS, and JAK inhibitor AG490 and PI3K inhibitor LY294002 could inhibit IL-27 induced CXCL9 release from RA-FLS (Figure 7).

## Discussion

To investigate the immunopathological roles of inflammatory cytokine IL-27 on joint inflammation, we first confirm the significantly elevated plasma level of IL-27 in RA patients than normal controls. Apart from immune effector cells, recent study showed that IL-27 can also activate non-immune effector cells such as keratinocytes [25]. Our present expression analysis of IL-27 receptor complex has suggested that IL-27 could activate other non-immune cell targets such as joint FLS. Human FLS constitutively expressed functional IL-27 receptor complex WSX-1 and gp130, and STAT1 was phosphorylated upon IL-27 stimulation (Figures 1 and 6). The present results are therefore in concordance with the expression of gp130 and WSX-1 on synovial biospsies [26-28]. IL-27-mediated activation effects have been shown to be mainly through the regulation of STAT1 phosphorylation [13,25,29]. For example, IL-27 could activate STAT1 to induce IL-12Rβ2 expression in naive CD4+T cells for Th1 differentiation [13]. IL-27-induced STAT1 activation was associated with proinflammatory effects in human monocytes and keratinocytes [25,29]. To further explore the difference of the activating effects of IL-27 on RA and control FLS, we performed functional and underlying mechanistic studies.

In the present study, we found that IL-27 was able to induce significantly higher expression of ICAM-1 and VCAM-1 and to augment TNF-α- and IL-1β-induced ICAM-1 and VCAM-1 on RA-FLS than that of control FLS (Figures 4 and 5), thus providing a novel immunopathological mechanism of IL-27 mediated joint inflammation in RA. ICAM-1 and VCAM-1 are transmembrane glycoprotein of the immunoglobulin supergene family expressed on many cell types. ICAM-1 and VCAM-1 participate in leukocyte-leukocyte, leukocyte-endothelium, and leukocyte-epithelium interactions and transendothelial migration [30]. Expression of VCAM-1 on synovial fibroblasts is also a clinical hallmark of RA [31]. Expression of these adhesion molecules on FLS has been implicated in the pathogenesis inflammation in RA by the induction of the infiltration of leucocytes into local joint [32]. Because of the pivotal role of ICAM-1 and VCAM-1 in inflammation, antisense oligonucleotides, monoclonal antibodies or blockage compound against ICAM-1 and VCAM-1 have been demonstrated with therapeutic beneficial effects in inflammatory diseases [33]. Expression of ICAM-1 and VCAM-1 on epithelial cells can be up-regulated by cytokines such as TNF-α, IFN-γ or IL-1δ [34,35]. We herein provided evidence that IL-27 is a potent inducer of ICAM-1 and VCAM-1, especially in combination with TNF-α or IL-1δ, on RA-FLS but not control FLS, thereby elucidating the selective pathological roles of IL-27 in RA.

A panel of inflammatory cytokines such as TNF-α, IL-1, IL-6, IL-23, and IL-2 families are active in the joints of the patients with RA by causing inflammation, articular destruction, and the comorbidities [24]. We also found that IL-27 could induce a significantly higher amount of IL-6 from RA-FLS than control FLS (*P *< 0.05, data not shown). To examine whether IL-27 also modulates the expression of chemokines in FLS, we found that IL-27 can induce significantly higher release of inflammatory chemokines CCL2, CXCL9 and CXCL10 from RA-FLS than control FLS. Combined treatment of IL-27 and TNF-α or IL-1β also resulted in the synergistic production of CCL5, CXCL9 and CXCL10 of RA-FLS (Figures 4 and 5). CCL2, CCL5, CXCL9 and CXCL10 are crucial inflammatory chemokines for the chemoattraction of Th1 cells and macrophages [36]. Those chemokines may also stimulate FLS to release other cytokines and MMP for the cartilage degradation and pannus formation [36]. In fact, we observed that IL-27 could induce the release of MMP-1 from RA-FLS but not control FLS. MMP-1 has been suggested to correlate with the invasive growth of FLS in RA [3]. Furthermore, CCL2, CCL5, CXCL9 and CXCL10 have been shown to be highly expressed in synovial fluid and synovial tissue [37-39]. Stimulation of RA-FLS with CCL2 and CCL5 can enhance the production of IL-6 and CXCL8 [40], while CCL2, CXCL10, CCL5 and CXCL9 induce the gelatinase and collagenase activities in the supernatants of cultured FLS [41]. CCL2 and CCL5 can stimulate MMP production by chondrocytes, inhibit proteoglycan synthesis and enhance proteoglycan release from the chondrocytes [42]. In addition, CCL5 can induce the expression of inducible nitric oxide synthase, IL-6 and MMP in chondrocytes [43]. FLS cells are a kind of primary cell without cell proliferation and division. The FLS cell numbers were constant during culture and therefore the constant cell number did not have any effect on the chemokines and MMP concentrations being measured in the cell culture supernatant.

Although IL-27 alone could not activate CCL5 release from RA and control FLS, IL-27 exhibited synergistic effect with TNF-α to induce CCL5 release in RA-FLS. Actually, TNF-α can induce the expression of CCL5 in cultured FLS [44]. Our results show that RA-FLS exhibits a lower level of TNF-induced ICAM-1 and VCAM-1 expression as compared to control FLS. It may be due to the lower cell surface expression of TNFRp55 (data not shown), the crucial TNF receptor component for the expression of adhesion molecule ICAM-1 and VCAM-1, on RA-FLS compared with control FLS [45,46]. However, our previous study showed that the synergistic activating effects of IL-27 and TNF-α on bronchial epithelial cells were partially due to the IL-27 up-regulated expression of TNF-α receptor p55TNFR [47]. IL-27 stimulation could also lead to the increased expression of TNF-α and IL-1β in primary human monocytes [14,48]. The above may account for the synergistic effect of IL-27, TNF-α and IL-1β for chemokine release. Since FLS, monocytes and Th1 cells play important roles in the pathogenesis of RA, the enhanced production of ICAM-1 and VCAM-1, and chemokines for macrophages and Th1 cells of RA-FLS induced by IL-27, TNF-α and IL-1β should contribute to the development of inflammation.

IL-27 has been reported to activate the JAK/STAT signaling pathway in T lymphocytes [49], but the signaling pathways of IL-27 in FLS have not been reported. We have previously reported the involvement of ERK, JNK, JAK and p38 MAPK pathways in the expression of adhesion molecules and release of cytokines and chemokines from activated bronchial epithelial cells upon exposure to diverse stimuli such as TNF-α, IL-4, IL-13 and IL-31 [50,51]. IL-27 stimulation could lead to receptor-mediated tyrosine phosphorylation of the STAT family [52]. Our study showed that incubation with IL-27 resulted in STAT1 tyrosine phosphorylation within five minutes and maintained it for 30 minutes in both control and RA-FLS, with a more potent activation in RA-FLS (Figure 6A). This discrepancy may account for the higher induction of adhesion molecules and release of chemokine upon IL-27 stimulation in RA-FLS. We also found that PI3K-AKT and JNK pathways were activated in both RA and control FLS in response to IL-27, whereas JAK-2 in RA-FLS was activated upon IL-27 activation (Figure 6). Actually, previous studies have revealed that JNK and AKT involve the inflammatory mechanisms of FLS in RA [53,54].

Using selective inhibitors that could suppress the activation of their corresponding signaling pathways, we further elucidated the involvement of different signaling pathways in regulating expression of adhesion molecules and chemokines of FLS. These inhibition experiments demonstrated that the IL-27-induced up-regulation of ICAM-1 on control FLS, and ICAM and VCAM-1 on RA-FLS was regulated by the activation of intracellular PI3K-AKT and JNK pathways (Figure 7). Regarding the chemokine release, IL-27 induced CXCL9 and CXCL10 from control FLS, CCL2 and CXCL10 from RA-FLS and CXCL9 from RA-FLS was found to be mediated by PI3K and JNK pathways, JAK, PI3K and JNK pathways, and JAK and PI3K pathways, respectively (Figure 7). The above discrepancy of the intracellular signaling mechanisms between RA and control FLS for the induction of adhesion molecules and chemokines indicates the distinct signaling pathways in inflammatory responses. Such induction of chemokines by IL-27 in RA-FLS was not completely inhibited by those inhibitors, other unidentified signaling pathways might therefore contribute to the chemokine expression. Further experiments may be required to investigate the regulatory mechanisms of different signaling molecules and transcription factors for the induction of chemokines in human FLS upon cytokine treatment.

Using a murine model, IL-27 can exhibit a suppressive effect on inflammation and can attenuate collagen-induced arthritis when administered at the onset of the disease via the blocking of Th17 differentiation from naïve T helper cells [17,55,56]. On the other hand, the late onset of IL-27 was unable to downregulate Th17 in established RA, thereby inducing the inflammation of the ongoing adjuvant-induced arthritis *in vivo *[57]. In view of that, our study using FLS derived from normal subjects and established RA patients, therefore, only exhibits the activating effects rather than the suppressive effects of IL-27. Since IL-27 is detected at significant concentrations in the joints of patients with ongoing RA, neutralization of IL-27p28 has been suggested as a novel therapy [57]. Previous therapeutic approaches relied on disease-modifying antirheumatic drugs (DMARDs) such as methotrexate and sulfasalazine that had only partial clinical beneficial effects and were associated with toxicity [24]. In view of the application of antibodies for the blockade of TNF-alpha, IL-1β and IL-6 receptor as treatments for RA, other cytokines such as IL-27 may therefore offer alternative targets for therapeutic intervention or may be useful as predictive biomarkers of established RA [57-59]. Because of the recent advances in the application of MAPK and NF-κB inhibitors as potential anti-inflammatory agents [60], our present study should also provide new clues for the development of novel treatment for IL-27-mediated RA inflammation.

## Conclusions

Our results indicate the differential inflammatory IL-27-mediated intracellular signaling mechanisms including STAT1, PI3K-AKT, JNK and JAK pathways for the activation of RA and control FLS. They suggest the distinct signaling pathways during inflammatory responses in RA. As a result, IL-27 plays crucial immunopathological roles in joint inflammation in RA by the induction of adhesion molecules, cytokines and chemokines, especially combined with TNF-α and IL-1β. Our study also provides a biochemical basis for the development of a new modality for RA.

## Abbreviations

CBA: cytometric bead array; DMARDs: disease-modifying antirheumatic drugs; DMSO: dimethyl sulfoxide; EBI3: EBV-induced protein 3; ERK: extracellular signal-regulated protein kinase; ESR: erythrocyte sedimentation rate; FLS: fibroblast-like synoviocytes; ICAM: intercellular adhesion molecule; IFN: interferon; IL: interleukin; JAK: Janus kinase; JNK: c-Jun amino-terminal kinase; MAPK: mitogen activated protein kinases; MFI: mean fluorescence intensity; MMPs: matrix metalloproteinases; PI3K: phosphatidylinositol 3-OH kinase; RA: rheumatoid arthritis; RANKL: receptor activator of nuclear factor-κB ligand; STAT: signal transducers and activators of transcription; TCCR: T cell cytokine receptor; Th: T helper; TNF: tumor necrosis factor; VCAM: vascular cell adhesion molecule

## Competing interests

The authors declare that they have no competing interests.

## Authors' contributions

CKW and CWKL designed the study and secured funding for it. DPC performed the experiments and processed the data. LST and EKL designed the study, recruited the patients, and acquired and analyzed the clinical data. DPC and CKW analyzed and interpreted the data. CKW, DPC and YBY prepared the manuscript. All authors read and approved the final manuscript.
